# Iron-deficiency anemia reduces cardiac contraction by downregulating RyR2 channels and suppressing SERCA pump activity

**DOI:** 10.1172/jci.insight.125618

**Published:** 2019-04-04

**Authors:** Yu Jin Chung, Antao Luo, Kyung Chan Park, Aminah A. Loonat, Samira Lakhal-Littleton, Peter A. Robbins, Pawel Swietach

**Affiliations:** 1 Department of Physiology, Anatomy and Genetics, University of Oxford, Oxford, United Kingdom.; 2 Medical College, Wuhan University of Science and Technology, Wuhan, China.

**Keywords:** Cardiology, Calcium signaling, Excitation contraction coupling, Heart failure

## Abstract

Iron deficiency is present in ~50% of heart failure (HF) patients. Large multicenter trials have shown that treatment of iron deficiency with i.v. iron benefits HF patients, but the underlying mechanisms are not known. To investigate the actions of iron deficiency on the heart, mice were fed an iron-depleted diet, and some received i.v. ferric carboxymaltose (FCM), an iron supplementation used clinically. Iron-deficient animals became anemic and had reduced ventricular ejection fraction measured by magnetic resonance imaging. Ca^2+^ signaling, a pathway linked to the contractile deficit in failing hearts, was also significantly affected. Ventricular myocytes isolated from iron-deficient animals produced smaller Ca^2+^ transients from an elevated diastolic baseline but had unchanged sarcoplasmic reticulum (SR) Ca^2+^ load, trigger L-type Ca^2+^ current, or cytoplasmic Ca^2+^ buffering. Reduced fractional release from the SR was due to downregulated RyR2 channels, detected at protein and message levels. The constancy of diastolic SR Ca^2+^ load is explained by reduced RyR2 permeability in combination with right-shifted SERCA activity due to dephosphorylation of its regulator phospholamban. Supplementing iron levels with FCM restored normal Ca^2+^ signaling and ejection fraction. Thus, 2 Ca^2+^-handling proteins previously implicated in HF become functionally impaired in iron-deficiency anemia, but their activity is rescued by i.v. iron supplementation.

## Abstract 2

Iron deficiency, a common comorbidity in heart failure, reduces the strength of cardiac contraction through effects on Ca2+-handling proteins crucial for normal cardiac function but typically deranged failing hearts.

## Introduction

Iron deficiency is the most common medical disorder in the world (1, 2), and around half of all patients with heart failure (HF) are iron deficient (3–5). A number of large, multicenter trials of i.v. iron administration in HF have demonstrated substantive patient benefit (6–8), but the mechanism by which this occurs is unknown. In mice, severe iron deficiency produced by genetic deletion of the transferrin receptor gene results in fatal cardiac dysfunction (9). Here, we sought to determine whether more modest degrees of iron deficiency also result in cardiac dysfunction and, if so, what changes underlie such dysfunction.

Iron could affect cardiac function through myriad pathways. These include alterations in gene expression through iron- and oxygen-dependent enzymes, such as prolyl hydroxylases (PHDs) and lysine demethylases (KDMs), which regulate hypoxia-inducible factor (HIF) (10, 11) and histone methylation marks (12, 13), respectively. Alternatively, iron may exert more acute effects on the heart through changes in metabolism (14). In terms of specific protein targets in the heart, there is a general consensus that much of the contractile deficit in HF is due to an attenuation of cardiomyocyte Ca^2+^ signals (15), but the effect of iron deficiency on this critically important pathway is unknown. Ca^2+^-handling proteins in the ventricular myocyte include L-type voltage-gated Ca^2+^ channels (LCC) Na^+^/Ca^2+^ exchangers (NCX), ryanodine receptor channels (RyR2), and Ca^2+^ ATPase pumps at the sarcoplasmic reticulum (SERCA) and plasmalemma (PMCA). During electrical excitation, LCC conduct a current of Ca^2+^ ions, which triggers RyR2 channels at the sarcoplasmic reticulum (SR) to release stored Ca^2+^ ions, producing a cytoplasmic Ca^2+^ transient (CaT). This signal is terminated by Ca^2+^ reuptake into the SR by SERCA and extrusion from the cell by NCX and PMCA.

The aim of this study was to investigate whether iron deficiency can adversely affect cardiac function and Ca^2+^ signaling in an otherwise healthy organism. For this purpose, we established iron deficiency in mice by restricting their dietary iron intake and monitored the ensuing changes in iron status and cardiac function. To explore the effects of subsequent iron repletion, some animals were supplemented i.v. with ferric carboxymaltose (FCM), a formulation used clinically in humans (6). The dietary intervention produced iron deficiency and anemia, which were reversed by FCM. By imaging cardiac contraction in vivo and cardiomyocyte Ca^2+^ signaling in vitro, we demonstrate that iron deficiency reduces ejection fraction (EF) by decreasing CaT amplitude. This systolic dysfunction relates to a downregulation of RyR2 channels and suppression of SERCA Ca^2+^ pumps, 2 proteins located at the SR membrane. I.v. iron supplementation restored normal CaT amplitude and EF. Thus, we propose that iron deficiency can lead to a loss-of-function of 2 Ca^2+^-handling proteins, commonly implicated in failing hearts (15). The restorative effect of FCM on CaTs offers a mechanistic explanation for the beneficial effect of iron supplementation on the heart.

## Results

### Establishing iron-deficiency anemia in mice.

**Figure 1:**
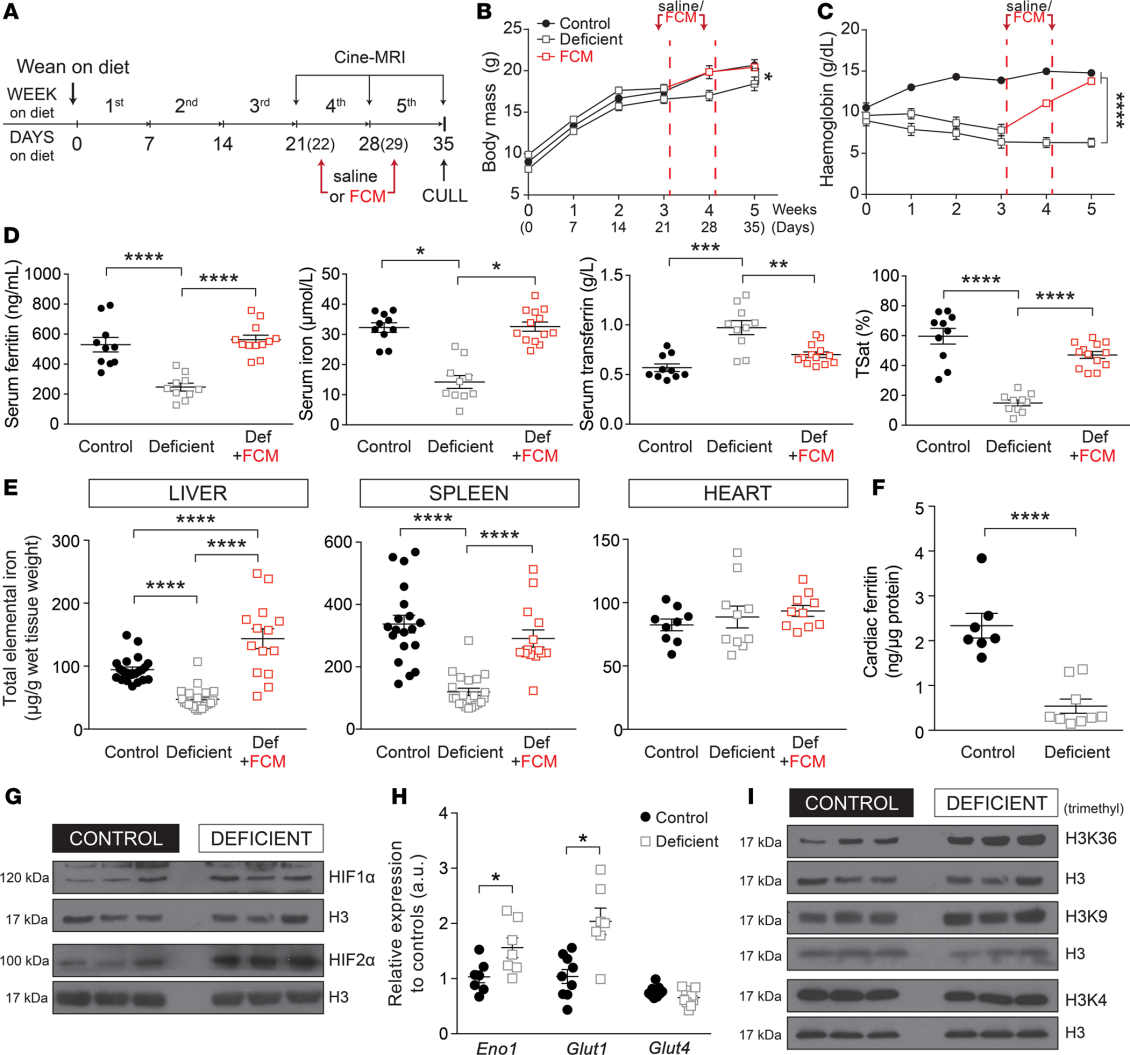
Characterizing the murine model of iron deficiency. (**A**) Timeline of the experimental protocol for establishing dietary iron deficiency in 3-week-old mice, showing time points at which either FCM or saline is injected, MRI scanning is performed, and hearts are excised for further studies. (**B**) Body mass and (**C**) tail vein blood hemoglobin concentration were measured during the 5-week dietary intervention (*n* = 6 animals/group). (**D**) Ferritin, iron, transferrin, and transferrin saturation (TSat) measured in the serum after 5 weeks of diet. *n* > 10 animals/group. (**E**) Elemental iron content in lysates of the liver, spleen, and heart, normalized to wet tissue weight, after 5 weeks of diet. *n* > 10 animals/group. (**F**) Cardiac ferritin expression measured by ELISA after 5 weeks of diet (*n* > 7 animals/group). (**G**) Immunoblot for HIF1α and HIF2α in cardiac lysates, normalized to histone H3 as the loading control, after 5 weeks of diet (*n* = 3 animals/group). (**H**) RT-qPCR analysis of mRNA levels of the HIF target genes *Eno1* and *Glut1* and non-HIF target gene *Glut4* after 5 weeks of diet. *n* = 9 animals/group. (**I**) Immunoblot for the histone marks H3K36, -K9, and -K4 after 5 weeks of diet (*n* = 3 animals/group). See Supplemental Table 1 for details of the number of experimental repeats. Note that HIF1α in **G** and H3K36me3 in **I** were blotted from the same membrane and, therefore, have the same nuclear loading control (histone H3). *P < 0.05, **P < 0.01, ***P < 0.001, ****P < 0.0001. *P* values determined by 2-way ANOVA for **A** and **B**; 1-way ANOVA for **D** and **E**; unpaired Student’s *t* test (2-tailed) for **F** and **H**.

A murine model of iron deficiency was established by weaning 3-week-old mice on an iron-deficient diet containing only 2-5 ppm iron for 5 weeks. To investigate the effect of iron supplementation, FCM (15 mg Fe/kg body mass) or saline (sham) were injected i.v. at 22 and 29 days of commencing the diet (Figure 1A). Body mass (Figure 1B) and blood hemoglobin (Figure 1C) were measured weekly. Animals on the iron-deficient diet had significantly lower hemoglobin (<70 g/l) but recovered to age-matched–control levels after 2 injections of FCM.

After 5 weeks of experimental diet, animals were culled to assess body iron reservoirs. Hematocrit was reduced from 46% ± 1% to 33% ± 1% (*n* = 4). Mean red blood cell volume (MCV), measured by flow cytometry in blood samples diluted in HEPES-buffered salt solution, was reduced from of 61.0 ± 0.5 fl to 50.8 ± 0.2 fl (*n* = 4). In animals on the iron-deficient diet, serum ferritin, serum iron, and transferrin saturation were reduced to 47%, 44%, and 25% of control, respectively, and total serum transferrin was raised to 171% of control; these indices returned to normal levels after 2 injections of FCM (Figure 1D). Dietary iron restriction also reduced total iron in the spleen (3-fold) and liver (2-fold). Two doses of FCM restored splenic levels to normal and overshot hepatic stores to 140% of controls (Figure 1E). Whereas total elemental iron in cardiac tissue was unaffected by the iron-deficient diet (Figure 1E), ferritin content was reduced in the hearts of iron-deficient mice (Figure 1F). This observation suggests that cardiac iron handling is affected in response to systemic iron deficiency.

To explore whether anemia affected oxygenation status in the heart, cardiac tissue was assessed for markers of hypoxia (10, 11). Hearts from iron-deficient animals had increased HIF expression (Figure 1G) and upregulation of HIF-target genes *Glut1* and *Eno1*, but not *Glut4*, a HIF-independent gene (Figure 1H). Another marker of oxygen/iron status is histone methylation, regulated in part by the iron-dependent dioxygenase JumonjiC KDMs. The demethylase KDM4A (12, 13), previously implicated in HF (16), acts specifically on di- and trimethylated H3K36 and H3K9 (13), which were found to be enriched in the hearts of iron-deficient animals (Figure 1I and Supplemental Figure 1; supplemental material available online with this article; https://doi.org/10.1172/jci.insight.125618DS1). These histone marks are associated with activation (H3K36me3) and repression (H3K9me3) of transcription (17) and are known to alter gene expression in experimental models of cardiac dysfunction (16–19). In contrast, H3K4, the substrate for KDM5, had an unaltered trimethylated state (Figure 1I).

In summary, mice on the dietary regime have iron-deficiency anemia that falls in the range reported in anemic HF patients and triggers markers of hypoxia and iron depletion in cardiac tissue.

### Cardiac contractile dysfunction in iron-deficiency anemia.

**Figure 2:**
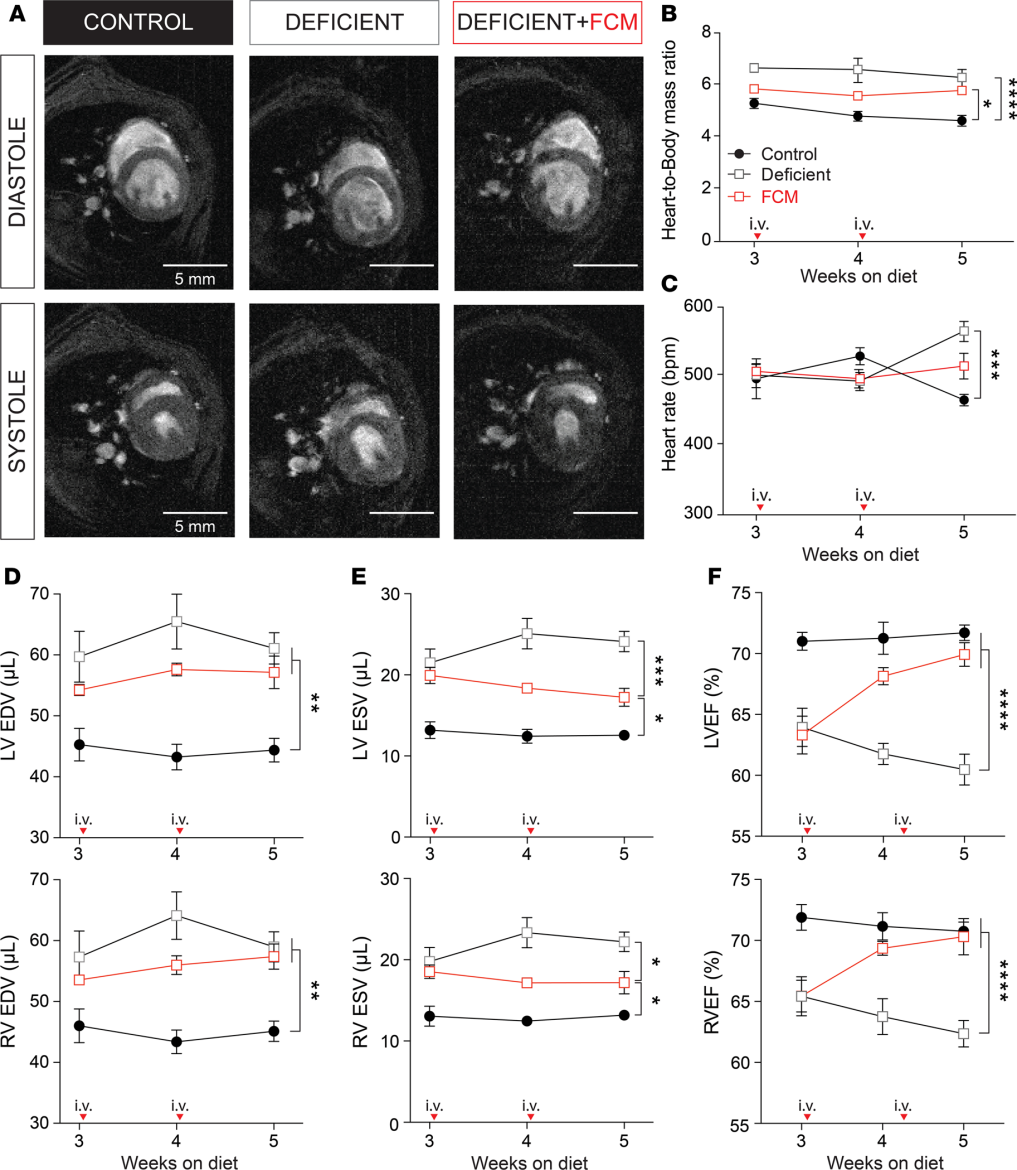
Effects of iron-deficiency anemia on cardiac function. (**A**) Representative Cine-MR images obtained from control, deficient, and iron supplemented animals scanned after 5 weeks of diet, showing 1 slice (out of 11 collected in the *z* axis) at systole and diastole of 1 exemplar cardiac cycle. (**B**) Heart/body mass ratio and (**C**) heart rate. Left and right ventricular (**D**) end-diastolic volume (EDV), (**E**) end-systolic volume (ESV), and (**F**) ejection fraction (LVEF, RVEF) calculated from volume-rendering of MR images (*n* = 6 animals/group). Arrows indicate point at which FCM was injected. *P < 0.05, **P < 0.01, ***P < 0.001, ****P < 0.0001. *P* values were determined using unpaired Student’s *t* test (2-tailed).

To measure the effect of iron-deficiency anemia on cardiac performance in vivo, cine-MRI was performed on days 21, 28, and 35 (Figure 2A). Over the course of the experiment, heart/body mass ratio was highest in iron-deficient animals, an effect that did not return to control levels even after 2 injections of FCM (Figure 2B). The larger heart/body mass ratio in iron-deficient animals may relate to a combination of factors, including their smaller body size (Figure 1B). Heart rate was not different between the 3 experimental groups until week 5 of the diet, at which point iron-deficient animals became modestly tachycardic (21% increase) (Figure 2C). For the duration of the dietary regime, iron-deficient animals had higher left ventricular (LV) and right ventricular (RV) end-diastolic volumes (EDV), a difference that was not fully restored by FCM (Figure 2D). The LV and RV end-systolic volumes (ESV) were significantly higher in iron-deficient animals, indicating a less complete emptying of the ventricles (Figure 2E). Ventricular emptying improved progressively with consecutive FCM supplementation. Regarding LVEF and RVEF, contractile performance progressively worsened under continued dietary iron restriction (15% decrease by week 5) but improved after 1 dose of FCM and returned to normal levels after the second FCM injection (Figure 2F). Thus, contractile performance was moderately impaired in iron-deficiency anemia, and iron supplementation was able to fully restore EF through a compensatory increase in stroke volume.

### Iron-deficiency anemia reduces CaT amplitude.

**Figure 3:**
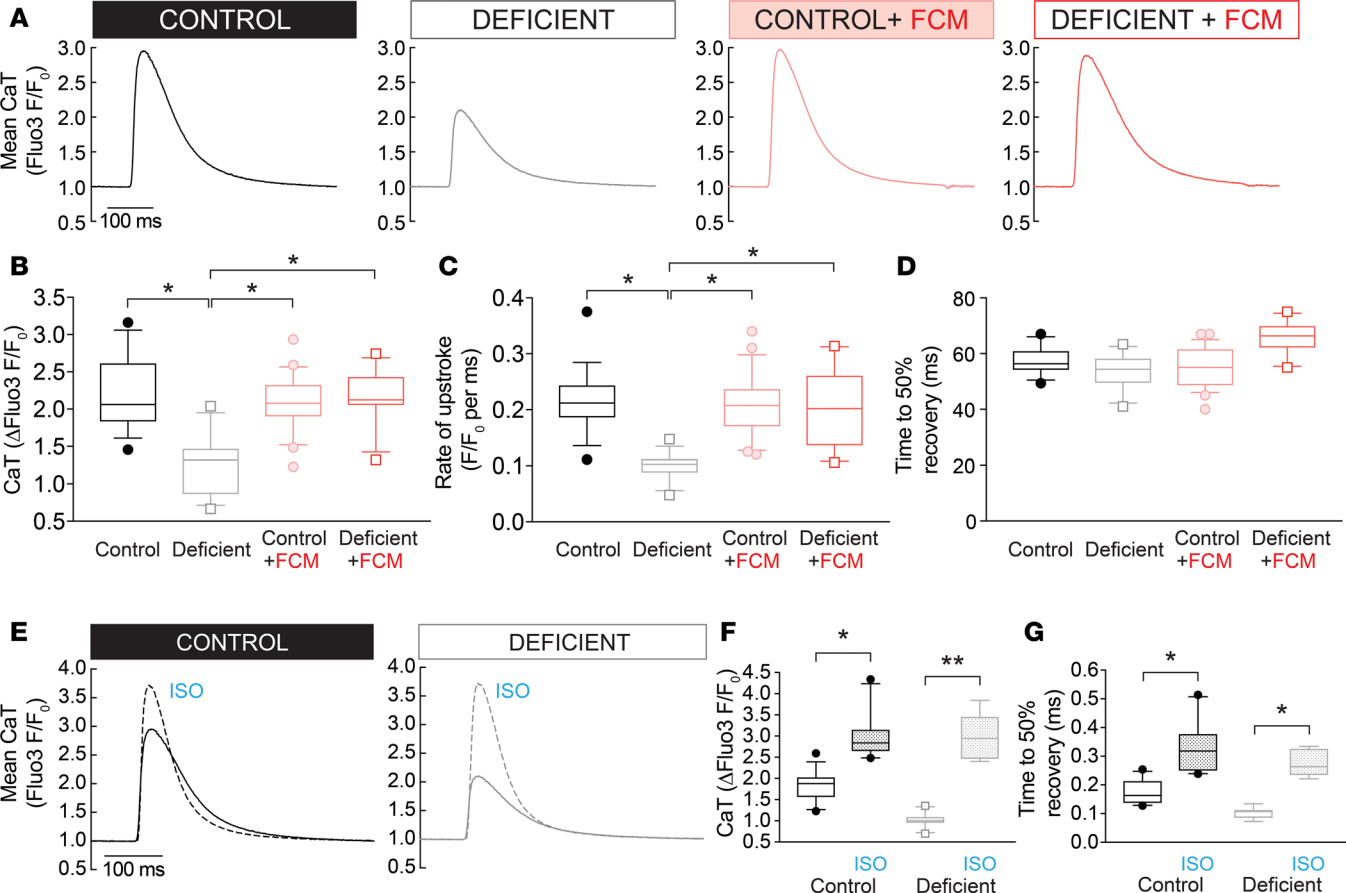
Effects of iron deficiency on myocyte Ca^2+^ signals. (**A**) Ca^2+^ transients (CaT) were measured in 2 Hz electrically paced myocytes loaded with Fluo3 and imaged in line scan mode (*n* > 20 cells from 3 animals/group; error bars not included for clarity). CaTs were analyzed in terms of (**B**) amplitude, (**C**) rate of upstroke measured to the half-maximal point, and (**D**) time to 50% recovery from the systolic peak (*n* > 20 cells from 3 animals/group). (**E**) CaTs recorded in myocytes stimulated acutely with 100 nM isoproterenol (ISO; dashed line). In the presence of ISO, CaT amplitude (**F**) was no different between control and iron-deficient groups, and time to 50% recovery (**G**) was accelerated (*n* > 20 cells from 3 animals/group). See Supplemental Table 1 for details of the number of experimental repeats, and see Supplemental Table 2 for details of nested (hierarchical) 1-way ANOVA analyses performed for data shown in **B–D** and **F–G**. *P < 0.05, **P < 0.01.

The time course of electrically evoked CaTs was determined in myocytes loaded with the fluorescent Ca^2+^ reporter Fluo3 and imaged in line-scan mode for high temporal resolution (Figure 3A). Field stimulation at 2 Hz produced substantially smaller CaTs in myocytes from iron-deficient mice (67% of control), but in animals injected with FCM, CaT amplitude was restored to control levels (Figure 3B). In contrast, i.v. iron supplementation in animals on the control diet had no additive effect on the CaT time course, arguing against a direct inotropic effect of FCM. The slope of the fluorescence-signal rise during the CaT upstroke (measured up to the half-maximal point) was slowed by 34% in iron-deficiency anemia and was restored fully by iron supplementation (Figure 3C). Recovery from systolic [Ca^2+^] levels is often quantified by the time taken to decay to 50% fluorescence; this index was not affected in iron-deficiency anemia (Figure 3D). It is, however, important to note that this index does not provide a complete appraisal of SERCA activity.

Next, CaTs were measured in myocytes treated acutely with isoproterenol (ISO; 100 nM), a β-agonist, to interrogate Ca^2+^ handling under strong stimulation by the cAMP/PKA pathway (Figure 3E). Under these conditions, differences in CaT amplitude were no longer apparent between iron-deficient and control animals (Figure 3F). The systolic level attained in these experiments did not saturate the dye, as even higher F/F_0_ ratios could be attained in separate calibration experiments (Supplemental Figure 2A). As confirmation that ISO treatment had engaged cAMP/PKA signaling, CaT recovery time was hastened in both groups of animals (Figure 3G). These findings may indicate that at least part of the effect of iron deficiency on Ca^2+^ handling involves a change in the activation state of proteins.

### Myocyte size, surface area, and dyadic organization are unchanged in iron deficiency.

**Figure 4:**
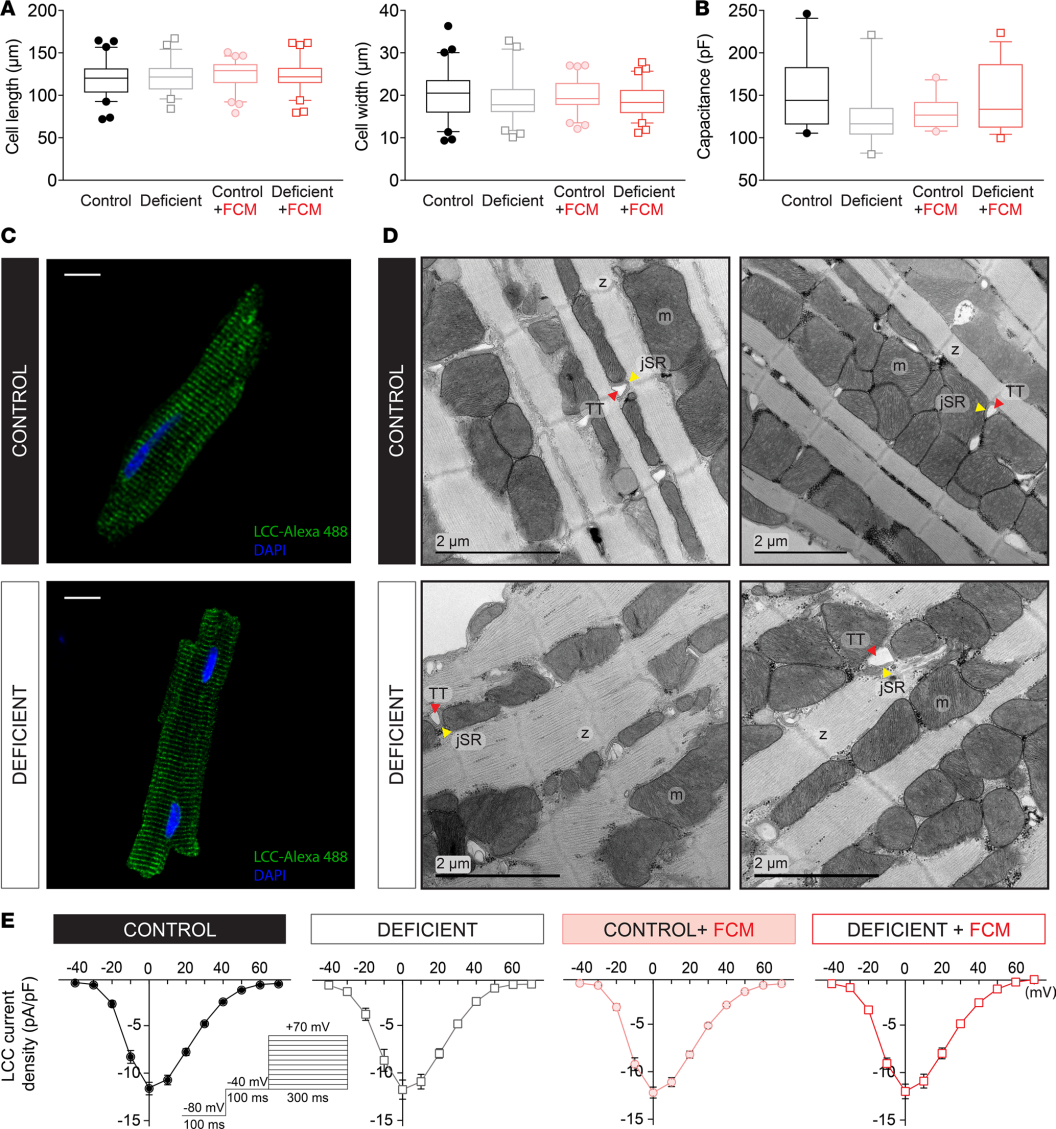
Effects of iron deficiency on myocyte geometry, dyadic structure, and Ca^2+^ currents. (**A**) Cell length and width measured in cSNARF1-loaded myocytes isolated from hearts (*n* = 120–140 cells from 4 animals/group) and (**B**) membrane capacitance measured in voltage-clamped myocytes (*n* > 15 cells from 4 animals/group). No difference in cell size or surface area were detected in myocytes from iron-deficient animals. (**C**) Immunofluorescence staining for L-type calcium channel (LCC) protein in permeabilized myocytes. LCCs are found predominantly in T-Tubules, and the staining pattern visualizes the state of sarcolemmal invaginations. No evidence of detubulation is observed in myocytes from iron-deficient mice. Scale bar: 20 μm. Exemplar images are shown. (**D**) Electron micrograph of isolated ventricular myocytes. Exemplar images are shown from *n* = 3 animals/group. TT, T-Tubule; jSR, junctional SR; m, mitochondria; z, Z-line. No changes in the dyadic ultrastructure were observed in myocytes from iron-deficient animals. (**E**) L-type Ca^2+^ current density, measured as a function of holding potential (*n* > 15 cells from 4 animals/group), by voltage-clamp electrophysiology. No difference in trigger Ca^2+^ current was observed in the 4 experimental groups. See Supplemental Table 1 for details of the number of experimental repeats.

There is now ample evidence for dysregulated cardiac Ca^2+^ handling in conditions involving maladaptive hypertrophic growth (15, 20–22). To investigate whether iron-deficiency anemia produced a substantial change in myocyte growth, cell dimensions were visualized in the xy plane by imaging cells loaded with carboxy SNARF1 (cSNARF1), a fluorescent dye with strong emission. Cell length and mean width were not significantly different in any of the 4 treatment groups (Figure 4A), implying that major hypertrophic remodeling had not occurred. The sarcolemma, in which ion channels and carriers are embedded, normally invaginates to form transverse tubules (T-Tubules), and various forms of HF have been linked with detubulation (23). To investigate if the surface membrane, which includes T-Tubules, becomes disrupted in iron-deficiency anemia, myocytes were voltage clamped to measure capacitance. Membrane capacitance was not different in any of the treatment groups (Figure 4B), indicating that sarcolemmal surface remained intact. Immunofluorescence staining for L-type Ca^2+^ channels, which are distributed mainly in T-Tubules (24), revealed a similar pattern in myocytes from control and iron-deficient mice (Figure 4C), implying against detubulation. The changes in CaTs observed in iron deficiency may have arisen from an ultrastructural remodeling of the dyadic space (e.g., a disruption in morphology of T-Tubules or distance between T tubules and junctional SR). This was investigated by electron microscopy, and exemplar images are shown in Figure 4D. From these, it can be inferred that iron deficiency did not affect dyadic organization.

### L-type Ca^2+^ current is not affected by iron-deficiency anemia.

Reduced CaT amplitude reported in iron deficiency may be related to a smaller trigger Ca^2+^ current. LCC was measured in myocytes by a voltage-clamp protocol consisting of a brief depolarization to inactivate Na^+^ channels, followed by a series of holding-voltage maneuvers to map the current-voltage relationship. All 4 treatment groups had comparable LCC density (Figure 4E), implying that the decrease in contractile function in iron deficiency could not be explained by a change in the trigger Ca^2+^ current.

### Cytoplasmic Ca^2+^ buffering is not affected in iron-deficiency anemia.

**Figure 5:**
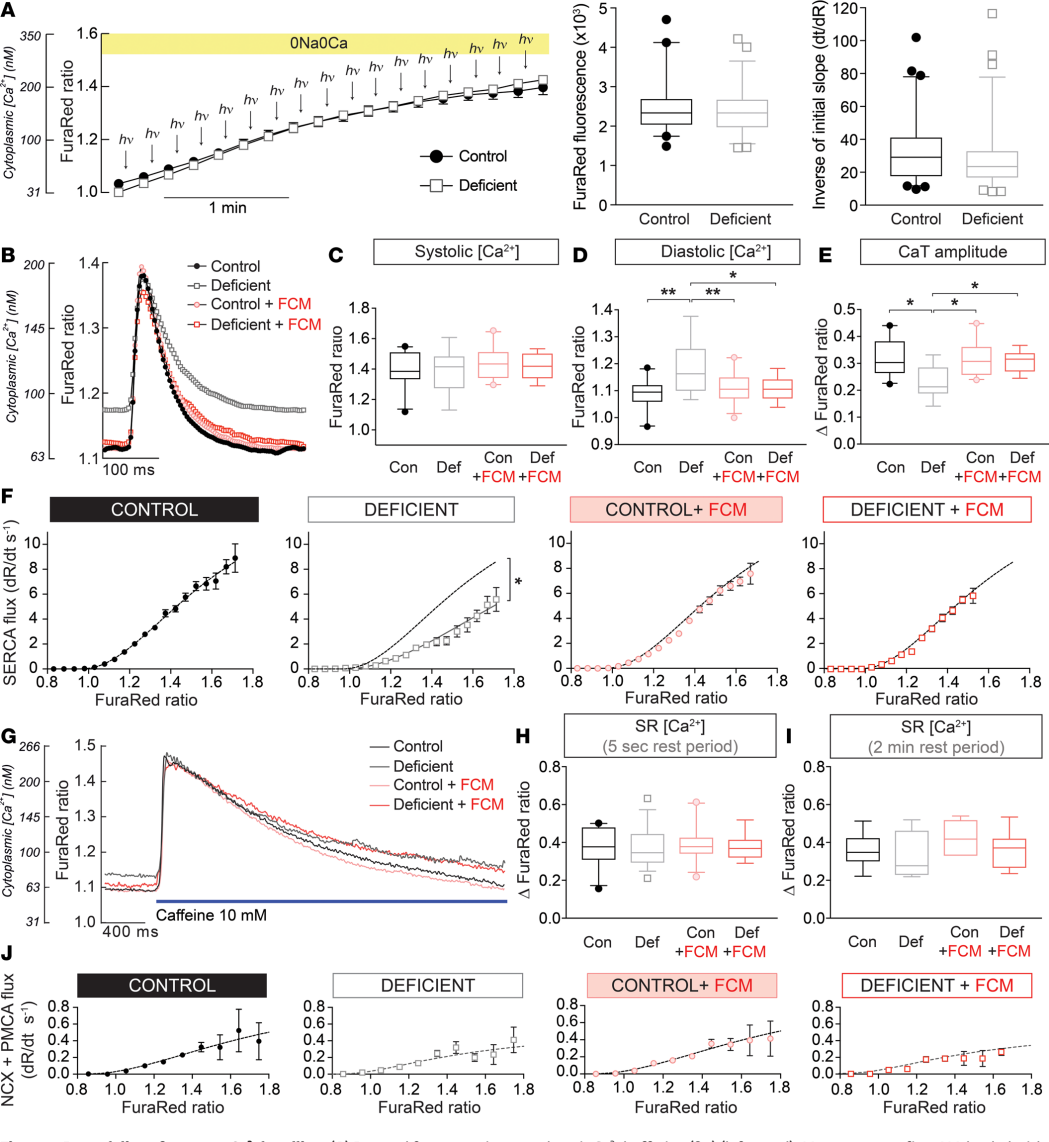
Remodeling of myocyte Ca^2+^ handling. (**A**) Protocol for measuring cytoplasmic Ca^2+^ buffering (β_Ca_) (left panel). Myocytes were first AM-loaded with FuraRed and the caged Ca^2+^-donor NP-EGTA, and they were subsequently superfused. After exposure to 10 mM caffeine (to empty SR), superfusate was switched to Na^+^-free/Ca^2+^-free (0Na0Ca) solution to block sarcolemmal Ca^2+^ fluxes. Photolytic uncaging and FuraRed imaging alternated in order to evoke and measure the rise in free [Ca^2+^] with FuraRed. The slope of the FuraRed time course provides inverse estimate of β_Ca_ (right panel). The extent of AM-ester loading was tested by FuraRed fluorescence near isobestic point (middle panel). Results from *n* = 30–35 cells from 3 animals/group. (**B**) Cells were electrically paced to evoke CaTs. Time courses show averaged CaTs measured from *n* > 30 cells from 4 animals/group. CaTs were analyzed in terms of (**C**) systolic [Ca^2+^], (**D**) diastolic [Ca^2+^], and (**E**) CaT amplitude. (**F**) The recovery from systolic Ca^2+^ was used to obtain the Ca^2+^-activation curve of SERCA, calculated from the rate of FuraRed ratio change after the peak of CaT (dashed line shows best fit for control group). (**G**) Approximately 5 seconds following a period of pacing, electrical stimulation was withdrawn and myocytes were rapidly exposed to a solution containing 10 mM caffeine delivered from a blunt micropipette. (**H**) The caffeine-evoked Ca^2+^ response was analyzed in terms of the peak Ca^2+^ level, which interrogates the SR load. (**I**) In separate experiments, the SR Ca^2+^ load was interrogated after a delayed delivery of caffeine (after 2-min rest period). (**J**) Ca^2+^-activation curves of NCX plus PMCA, calculated from the recovery of the caffeine-evoked cytoplasmic Ca^2+^ response. See Supplemental Table 1 for details of the number of experimental repeats and Supplemental Table 2 for details of nested (hierarchical) 1-wayANOVA analyses performed for data shown in **C–E** and **H–I**. *P < 0.05, **P < 0.01.

Buffering capacity (β_Ca_) is not routinely measured in studies of Ca^2+^ signaling, although it can affect the amplitude and time course of CaTs (25). β_Ca_ was assessed by recording the response of cytoplasmic [Ca^2+^], probed using a fluorescent dye, to a photolytic uncaging protocol that releases a fixed amount of Ca^2+^ ions. Myocytes were loaded with the membrane-permeant AM-ester of NP-EGTA (a caged Ca^2+^ substance) and FuraRed-AM (a ratiometric [Ca^2+^] reporter). A calibration curve for FuraRed is shown in Supplemental Figure 2, B–D. Importantly, saturating levels of [Ca^2+^] produce fluorescence ratios in excess of 2, and over the range between diastolic and systolic [Ca^2+^], the FuraRed ratio increases near-linearly with cytoplasmic [Ca^2+^]; thus, this dye is suitable for recording Ca^2+^ dynamics in myocytes. Ratiometric imaging eliminates potential artefacts due to photobleaching or dye loss, which would otherwise overestimate β_Ca_. To prevent Ca^2+^-induced Ca^2+^ release, the SR content was emptied with 10 mM caffeine, and to minimize sarcolemmal Ca^2+^ fluxes, myocytes were superfused in Na^+^-free/Ca^2+^-free solution (0Na0Ca). The measurement protocol (Figure 5A) was composed of whole-field excitation that alternated between FuraRed imaging (in dual-emission mode) and UV-photolytic uncaging. The rise in fluorescence ratio is proportional to the concentration of uncaged Ca^2+^ ions and inversely proportional to β_Ca_. Since the AM-loading protocol was executed consistently in all cells, the concentration of caged compound inside cells was comparable. In support of this, FuraRed fluorescence at its isosbestic point was the same in both groups. No significant difference in β_Ca_ was inferred between iron-deficient and control groups (Figure 5A).

### Iron deficiency raises diastolic Ca^2+^ and reduces fractional Ca^2+^ release from the SR.

In the absence of a change in trigger Ca^2+^ current or cytoplasmic Ca^2+^ buffering, the reduced CaT amplitude in myocytes isolated from iron-deficient mice is likely a result of reduced SR Ca^2+^ loading and/or decreased fractional release from the SR per excitation. This was investigated in myocytes AM-loaded with FuraRed, which provides a ratiometric read-out suitable for comparing levels of Ca^2+^ (e.g., diastolic and systolic) between different cells and conditions. Ratiometric imaging also allows transporter-generated Ca^2+^ fluxes to be plotted as Ca^2+^-activation curves, thus revealing any shifts in transporter kinetics. To optimize the dynamic range of FuraRed, imaging was performed in dual-excitation mode.

Two types of protocols were performed to interrogate Ca^2+^ handling. In the first protocol (Supplemental Figure 3A), a train of at least 10 CaTs was evoked by 2 Hz field stimulation and parameters were quantified once CaT amplitude attained steady state (Figure 5B). CaTs were analyzed in terms of systolic [Ca^2+^], diastolic [Ca^2+^], and CaT amplitude (Figure 5, C–E), as well as the Ca^2+^-activation curve of SERCA, which largely drives the recovery from systolic [Ca^2+^] (Figure 5F). Upon the cessation of electrical pacing, resting [Ca^2+^] was recorded, and then caffeine, a RyR2 activator, was released within 5 seconds from a blunt micropipette to evoke SR emptying (Figure 5G). The amplitude of the cytoplasmic [Ca^2+^] response to caffeine is an assay of SR content, and the subsequent recovery time course measures the combined activities of NCX and PMCA. The second protocol was designed to measure the extent of diastolic SR leak (Supplemental Figure 3B), which arises from the spontaneous opening of RyR2 channels and would reduce SR content and CaT amplitude. This leak was probed by measuring the response to caffeine applied after a longer, 2-minute period of rest, during which leaky RyR2 channels would have reduced the SR.

Myocytes isolated from iron-deficient mice had raised diastolic [Ca^2+^] but unchanged systolic [Ca^2+^] — hence, they had a smaller CaT amplitude (Figure 5E). The Ca^2+^-activation curve of SERCA was significantly right-shifted in myocytes from iron-deficient animals (Figure 5F). However, this fall in SERCA activity did not reduce the end-diastolic SR Ca^2+^ content (Figure 5H); instead, SR loading was unaffected by iron deficiency, indicating that the fractional release had decreased (i.e., a rebalancing of the pump-leak steady state). Lower fractional release is consistent with the slower upstroke of the CaT, measured with Fluo3 (Figure 3C), and explains the reduced CaT amplitude recorded with both Fluo3 (Figure 3B) and FuraRed (Figure 5E). These changes in Ca^2+^ handling were fully reversed in animals that received FCM i.v. Diastolic SR leak through RyR2 channels (Figure 5I) and Ca^2+^ extrusion by NCX and PMCA were not significant between the 4 treatment groups (Figure 5J). Taken together, the reduction in SR fractional Ca^2+^ release and CaT amplitude, taking place without a measurable change in SR Ca^2+^ load, can be explained by a suppression of SERCA activity combined with reduced RyR2 permeability.

### Remodeling of cardiac Ca^2+^ signals in iron-deficiency anemia relates to downregulated RyR2 channels and dephosphorylation of the SERCA regulator phospholamban.

**Figure 6:**
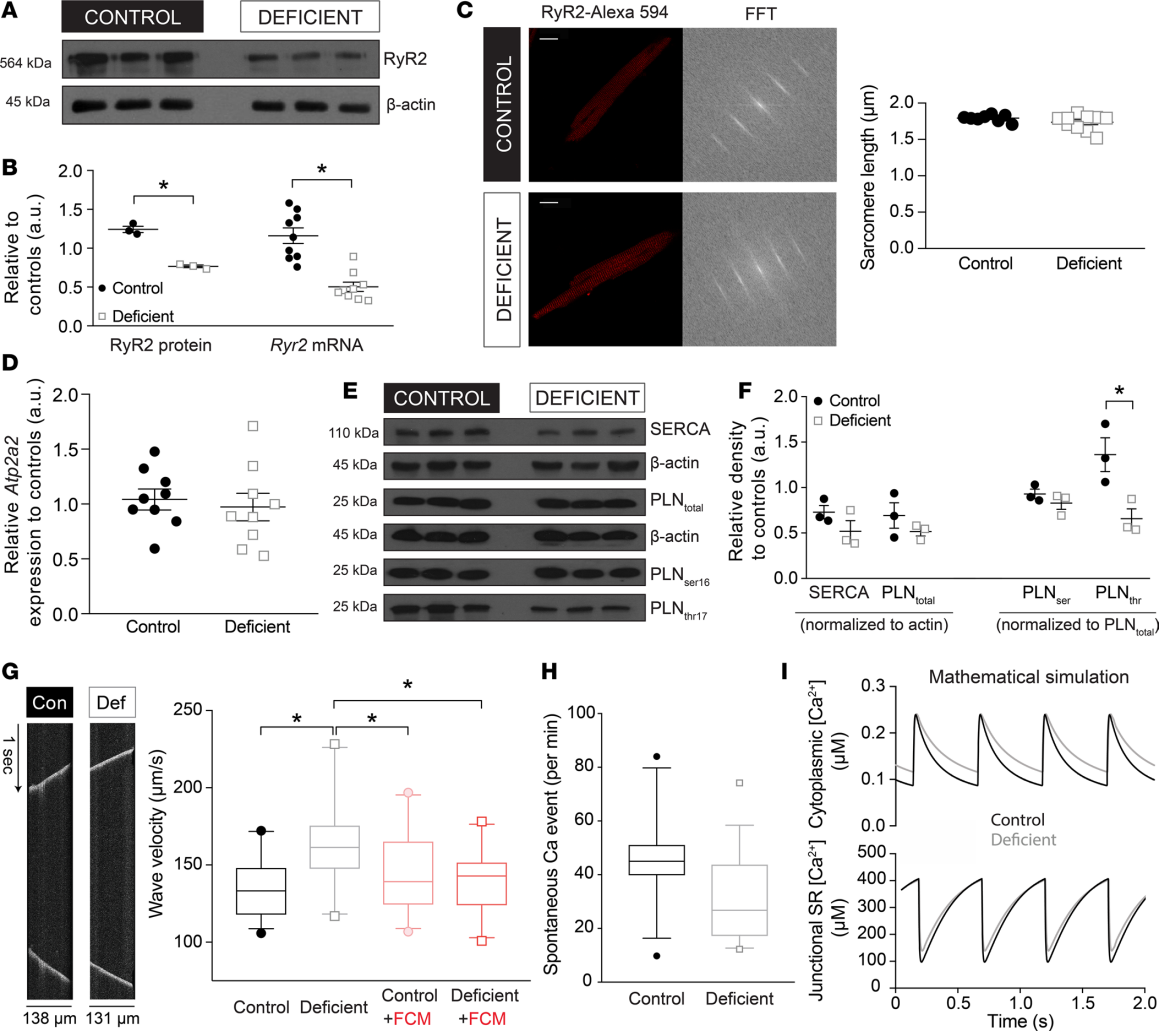
In iron-deficiency anemia, RyR2 is downregulated and SERCA activity is reduced by phospholamban dephosphorylation. (**A**) Immunoblot for RyR2 protein in cardiac lysates obtained from mice on a control or iron-deficient diet, showing downregulation in the latter. (**B**) Desitometric quantification of RyR2 immunoblot (left) and RT-qPCR analysis of *Ryr2* mRNA (right) showing decreased expression at message and protein level (*n* = 3 animals/group for immunoblot and 9 animals/group for qPCR). (**C**) Immunofluorescence staining for RyR2 in isolated and permeabilized adult mouse myocytes, alongside their fast Fourier transform (FFT) analyses for deriving sarcomeric spacing (*n* = 7 cells/group). Scale bar: 20 μm. (**D**) RT-qPCR analysis of mRNA level for the gene coding for SERCA (*Atp2a2*) (*n* = 9 animals/group), showing no difference in message level. (**E**) Immunoblot analysis of SERCA, total phospholamban (PLN_total_) and phospholamban phospho-Ser16 and phospho-Thr17 (PLN_ser16_, PLN_thr17_) performed on cardiac lysates obtained from mice on a control or iron-deficient diet (*n* = 3 animals/group). (**F**) Densitometric analysis of immunoblots. Immunoreactivity for SERCA and total PLN were normalized to actin, whereas phosphoproteins PLN_ser16_ and PLN_thr17_ were normalized to total PLN (*n* = 3 animals/group). Data are expressed relative to the control group. Iron deficiency produced a net dephosphorylation of PLN at Thr17. (**G**) Ca^2+^ waves were triggered by Ca^2+^-overloading adult mouse myocytes with superfusates of raised [Ca^2+^]. Ca^2+^ waves were recorded by line-scan along the length of cell, and their velocity was quantified from the angle of the waveform. Ca^2+^ waves propagated faster in myocytes from iron-deficient animals, consistent with reduced SERCA activity, which sets the diffusive spread of released Ca^2+^. (**H**) The incidence of spontaneous SR release events under conditions of Ca^2+^-overload, measured from line-scans. No significant change was found in the iron-deficient group. See Supplemental Table 1 for details of the number of experimental repeats and Supplemental Table 2 for details of nested (hierarchical) 1-way ANOVA analyses performed for data shown in **G** and **H**. (**I**) Results of the mathematical model of mouse myocyte Ca^2+^ signaling generated for control conditions (2 Hz pacing, 1 mM extracellular [Ca^2+^]) and iron deficiency conditions, featuring a 47% reduction in RyR2 permeability and a +0.2 μM right-shifted SERCA activity curve. To allow an offsetting of diastolic [Ca^2+^] to a higher level, PMCA and NCX activities were right-shifted by 1.4-fold. Time courses show the final CaT of a train of 200. *P < 0.05. *P* value determined by unpaired Student’s *t* test (2-tailed) for **B**, **D**, and **F**; nested (hierarchical) 1-way ANOVA for **G** and **H**.

Reduced RyR2 activity may be attributed to a downregulation of RyR2 expression. This was confirmed by the 2-fold reduction in RyR2 immunoreactivity and *Ryr2* message level measured in cardiac lysates from iron-deficient mice (Figure 6, A and B). Altered cardiac *Ryr2* expression in iron-deficient mice may affect RyR2 function by changing the distribution or organization of RyR2 protein. Indeed, previous studies have linked orphaned RyR2 proteins (i.e., channels expressed outside their normal Z-line locus) with HF (26). RyR2 channels normally assemble into clusters of 10–20 units along Z-lines where they are juxtaposed to LCCs (27). The downregulation of RyR2 may affect the spacing between clusters, which was inferred by fast Fourier transform analysis of RyR2 immunofluorescence images. This analysis showed no difference in the periodicity of RyR2 staining between the iron-deficient and control groups of myocytes (Figure 6C).

The reduction in SERCA-generated flux observed in myocytes from iron-deficient mice (Figure 5F) may relate to a downregulation of its gene, *Atp2a2*. However, at both message and protein level, there was no evidence for downregulation (Figure 6, D and E). Instead, the reduction in Ca^2+^ flux may relate to posttranslational changes. SERCA Ca^2+^ transport activity is strongly modulated by the state of phospholamban (PLN) phosphorylation (28). On Western blot, the stoichiometric relationship between SERCA and PLN appeared constant (Figure 6E). However, PLN phosphorylation at Thr17 (but not Ser16) was reduced 2-fold in iron-deficiency anemia (Figure 6F), which predicts a stronger inhibitory influence on SERCA (28). Physiologically (e.g., in response to sympathetic stimulation), PLN is phosphorylated sequentially at Ser16 and then Thr17 by PKA and calmodulin kinase II (CaMKII), respectively, producing an additive dysinhibition of the pump (28, 29). Previous studies have demonstrated Thr17 dephosphorylation during stop-flow ischemia (30, 31) due to reduced CaMKII activity (30), possibly relating to the tissue hypoxia.

Further interrogation of the spatial organization of RyR2 release sites and SERCA activity was done by measuring the properties of Ca^2+^ waves that propagate along the length of the myocyte as a result of a fire-diffuse-fire sequence involving adjacent RyR2 clusters. Ca^2+^ waves, imaged with Fluo3 in line-scan mode along the long axis of the myocyte, were triggered by raising superfusate calcium from 1–5 mM, which overloads the SR with Ca^2+^ (Figure 6G). Wave velocity, calculated from the angle of propagation on line scan, was accelerated in iron deficiency. As there was no difference in sarcomeric spacing, this observation can be explained by a slower reuptake of Ca^2+^ by SERCA pumps, allowing cytoplasmic Ca^2+^ ions to diffuse to more distant release sites.

Previous studies of cardiac pathologies have been linked to a remodeling of RyR2 channels and change in spontaneous release events, manifested by an increase in Ca^2+^ spark frequency (32–34). Although iron deficiency did not affect the magnitude of diastolic SR Ca^2+^ leak (Figure 5I), a change in the frequency of spontaneous SR Ca^2+^ release events remains plausible. To test this, Fluo3-loaded myocytes were Ca^2+^-overloaded by superfusion in 5 mM Ca^2+^, and release events were monitored over a period of 1 minute by line scan along the length of a myocyte. The frequency of all spontaneous release events registered in the line scan was not different between the iron-deficient and control groups (Figure 6H).

Considering the numerous feedback loops operating between Ca^2+^ handling proteins, it is difficult to predict intuitively the effect of altering one or more of these proteins on the overall CaT time course. For example, it is well established that changing RyR2 permeability alone has only a transient effect on CaTs because the ensuing rise in SR Ca^2+^ content offsets the reduced fractional release (35). Thus, to test if the observed RyR2 downregulation combined with the right-shift in the SERCA activation curve are sufficient to explain the decrease in CaT amplitude, a biophysical model of murine Ca^2+^ signaling (36) was run to simulate CaTs. To mimic the iron-deficient phenotype, SERCA K_m_ was increased (+0.2 μM) and RyR2 activity reduced (by 47%). Simulations were run in the CellML environment (http://www.cellml.com) for a test period of 100 seconds until a steady state amplitude was attained. As shown in Figure 6I, these parameter changes were sufficient to stably decrease CaT amplitude, without significantly affecting end-diastolic SR Ca^2+^ load. These simulations add further support to the proposed model of CaT remodeling in iron-deficiency anemia.

### The effect of hypoxia on Ca^2+^ signaling in cultured neonatal ventricular myocytes.

**Figure 7:**
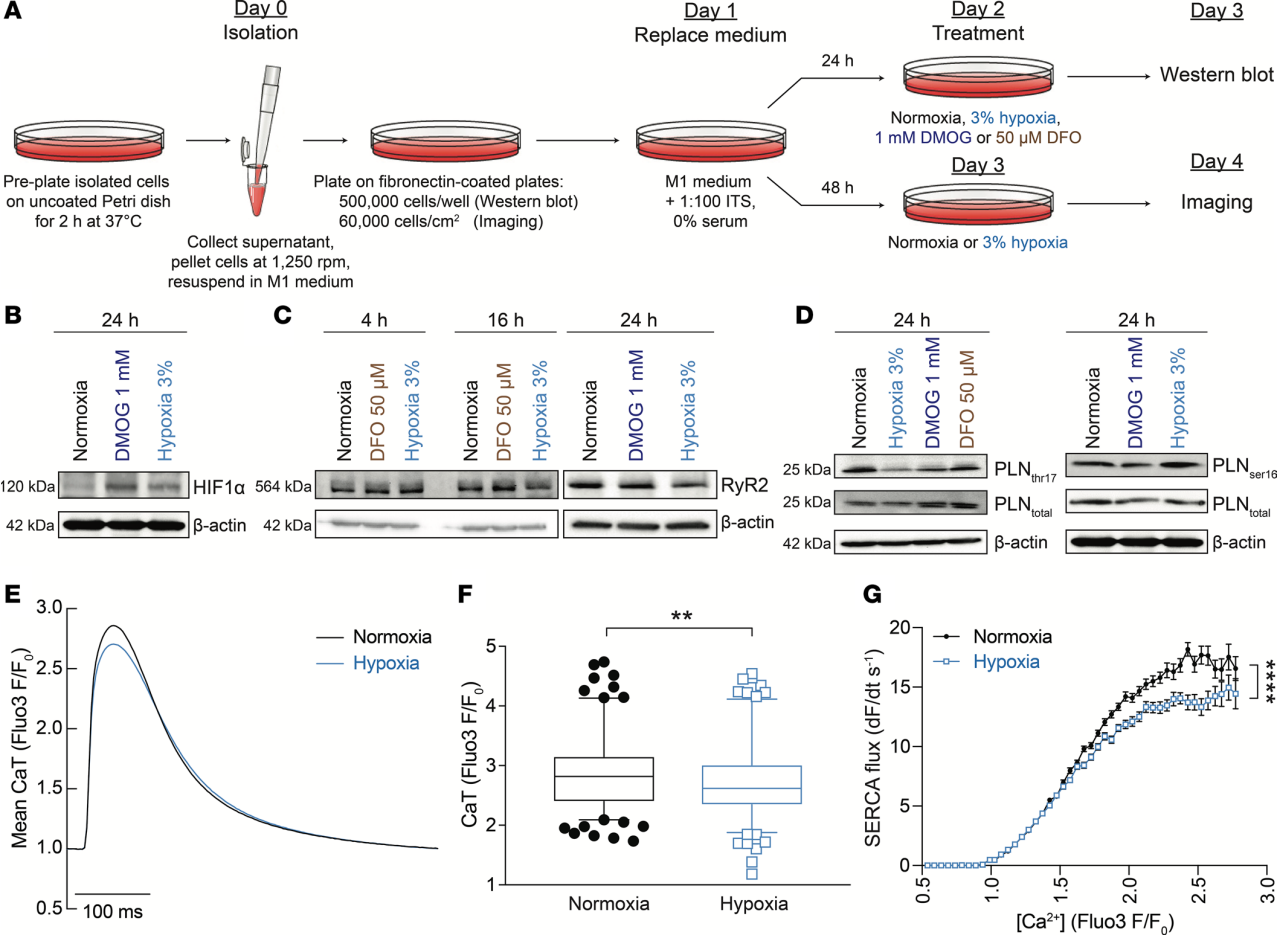
Ca^2+^ signaling in cultured neonatal ventricular myocytes. (**A**) Protocol showing the method of neonatal rat ventricular myocyte (NRVM) culture including the 24-hour period under experimental conditions. (**B**) Immunoblot of lysates prepared from NRVMs incubated in a normal atmosphere, under 3% oxygen (hypoxia) or in the presence of 1 mM DMOG for 24 hours, showing upregulation of HIF1α, consistent with the observed HIF induction in the hearts of mice with iron-deficiency anemia. (**C**) RyR2 immunoreactivity in lysates of neonatal ventricular myocytes was reduced in 3% hypoxia but not in 1 mM DMOG or 50 μM DFO (iron chelator). (**D**) Immunoblot for PLN and its phosphorylated forms at Thr17 (left panel) or Ser16 (right panel) performed on lysates prepared from neonatal rat ventricular myocytes incubated in a normal atmosphere, under 3% oxygen (hypoxia), in the presence of 1 mM DMOG or 50 μM DFO. Phosphorylation at Thr17 (but not Ser16) was reduced in 3% hypoxia. (**E**) Ca^2+^ transients measured in electrically paced, Fluo3-loaded myocytes cultured under normoxic (19% O_2_) or hypoxic (3% O_2_) conditions for 24 hours. The low-oxygen atmosphere in hypoxia-treated cells was maintained during imaging by placing an air flow chamber over cells and superfusing cells with N_2_-bubbled solutions. (**F**) Ca^2+^ transient amplitude was significantly reduced in hypoxia, according to hierarchical statistical analysis. (**G**) Flux, measured as the recovery from systolic Ca^2+^, was significantly slower in hypoxia (*n* = 180 cells from 5 litters for control and 173 cells from 5 litters for hypoxia). See Supplemental Table 3 for details of nested (hierarchical) 1-way ANOVA analyses. Note that HIF1α in **B** and PLN_ser16_ in **D** (blot shown on right) were blotted from the same membrane and, therefore, have the same loading control (β-actin). **P < 0.01, ****P < 0.0001.

In vivo, hypoxia and iron are strongly interrelated, as shown here by the effect of iron deficiency on markers of cardiac tissue hypoxia (Figure 1, G and H) and by previous studies showing a redistribution of iron toward BM in hypoxia (37). Thus, it is inherently difficult to determine whether iron deficiency in mice affects cardiac CaTs directly or via anemia. Some additional insight into the mechanism can be obtained from studying the response of myocytes under culture conditions, where it is more feasible to decouple the interplay between iron and hypoxia. The effect of iron depletion and hypoxia on cardiac proteins were investigated in vitro using neonatal rat ventricular myocytes (NRVMs), a preparation that can be cultured for extended periods of time (Figure 7A). Hypoxia at 3% was sufficient to modestly stabilize HIF (Figure 7B) and reduce RyR2 immunoreactivity after at least 16 hours of incubation (Figure 7C). Over this same timeframe, the iron chelator desferrioxamine (DFO; 50 μM) did not affect RyR2 expression, implying that tissue hypoxia may be playing the primary role in downregulating RyR2 in iron-deficient mice. The HIF-stabilizing drug dimethyloxaloylglycine (DMOG; 1 mM), applied for 24 hours, also did not evoke a downregulation of RyR2 immunoreactivity, implying that the hypoxic downregulation of RyR2 is not HIF mediated. Incubation in 3% hypoxia for 24 hours reduced PLN phosphorylation at Thr17 without affecting the state of Ser16 (Figure 7D). This change in phosphorylation was not observed in cells cultured with 1 mM DMOG or 50 μM DFO, suggesting an acute metabolic effect of oxygen, rather than a mechanism dependent on HIF signaling.

To test if these changes in Ca^2+^-handling proteins were sufficient to evoke a meaningful effect on Ca^2+^ signals in cultured myocytes, resembling the phenotype of iron-deficient mice, CaTs were triggered by electrical pacing (2 Hz) in Fluo3-loaded cells (Figure 7E). This dye produces stronger Ca^2+^-dependent signals than FuraRed due to considerably better cellular uptake. Hypoxia, which reduced RyR2 expression and PLN phosphorylation at Thr17, was maintained for a 24-hour period of incubation and then during measurements under superfusion by means of an air-flow chamber placed over the cells and by bubbling solutions with N_2_. Compared with normoxic controls, 3% hypoxia stably reduced the amplitude of CaTs (Figure 7F) and reduced the recovery rate from systolic levels (Figure 7G). Thus, hypoxia affects cardiac Ca^2+^ signals in a manner that resembles the changes observed in iron-deficiency anemia.

## Discussion

### Iron-deficiency anemia leads to a contractile deficit in the heart.

To study the effect of iron deficiency on the heart, we developed a model of dietary iron deficiency in otherwise healthy mice. The restricted iron intake falls short of the growing body’s demand, resulting in anemia, the mobilization of iron stores from the liver and spleen, and the emergence of markers of tissue hypoxia and iron depletion in the heart (Figure 1). Heart rate remained unaffected up to the fifth week of the diet, at which point iron-deficient animals developed modest tachycardia. From the third week of diet, iron-deficient animals had increased end-diastolic ventricular filling and less complete emptying at end-systole (Figure 2, D and E). Strikingly, iron-deficiency anemia produced a moderate reduction in LVEF and RVEF (Figure 2F), an effect that is not intuitively expected as a consequence of anemia. According to the Frank-Starling mechanism, the greater EDV (preload) in iron-deficient mice would be expected to produce a higher EF, but the opposite was measured. This decrease in EF was progressive (64% at 3 weeks and 60% at 5 weeks vs. 72% in age-matched controls), moderate in effect size (17%; *P* < 0.002), and resulted in an inadequate emptying of the ventricle during systole, as is characteristic of developing systolic dysfunction. Consistent with systolic dysfunction, myocytes isolated from iron-deficient mice fired smaller CaTs from an elevated diastolic level but reaching the same systolic peak as control animals, as determined using the ratiometeric Ca^2+^ dye FuraRed (Figure 5, C–E). These observations could not be made using nonratiometric reporters (e.g., Fluo3), as these do not normally report baseline levels; moreover, the routine mathematical operation of normalizing fluorescence to starting levels (F/F_0_) disguises any differences in diastolic [Ca^2+^]. The size of the SR Ca^2+^ store in diastole was unchanged in iron deficiency (Figure 5H), but its fractional release was reduced — hence, the smaller CaT amplitude and contraction.

Treatment of animals with i.v. FCM, a clinically approved iron formulation, reversed the anemia and restored normal EF and CaT amplitude. It is noteworthy that not all cardiac parameters were restored fully by FCM in the timeframe of this study (e.g., the changes in diastolic filling and heart/body mass ratio) (Figure 2). The effect of iron supplementation on Ca^2+^ signaling could be attributed to a restoration of adequate iron stores because FCM administered to animals on a normal diet had no effect on CaTs.

### Mechanisms of remodeled Ca^2+^ signaling.

Iron deficiency did not result in any detectable myocyte hypertrophy or detubulation (Figure 4, A–D), 2 examples of changes that can take place in failing hearts (23, 38). Furthermore, changes in the cytoplasmic Ca^2+^ signal occurred without a change in L-type Ca^2+^ current (Figure 4E), diastolic SR Ca^2+^ leak (Figure 5I), cytoplasmic Ca^2+^ buffering (Figure 5A), or the frequency of spontaneous Ca^2+^ release events (Figure 6H). Instead, the 2 proteins that underwent substantive remodeling were RyR2 channels and SERCA pumps.

The reduction in RyR2 activity was inferred from the rise-time of CaTs (Figure 3C) and fractional SR release (Figure 5, C–F), both of which are reduced in iron-deficient mice. RyR2 expression was reduced at both message and protein level (Figure 6, A and B) to an extent that could explain the functional deficit. RyR2 downregulation occurred without a change in spacing between RyR2-tethering Z-lines (Figure 6C), arguing against an ultrastructural derangement but, instead, suggesting a reduction in the number of channels per cluster. Stressors, such as pressure/volume overload, circulating hormones, cytokines, and reactive oxygen species, are known to trigger cardiac remodeling (39) through a transcriptional reprogramming of cardiac gene expression (40). Indeed, markers of hypoxia and iron depletion became apparent in the hearts of iron-deficient animals. Tissue hypoxia, a likely outcome of anemia, was demonstrated by the induction of HIF signaling in the heart (Figure 1, G and H). Iron deficiency, with or without anemia, can also influence the iron binding protein/iron response element (IRP/IRE) regulatory system, which, in turn, affects the translation and/or stability of mRNAs containing an IRE in the untranslated region (1). The activation of the IRP/IRE system is illustrated by the lower expression of cardiac ferritin protein (IRE in 5′ UTR, suggesting inhibition of translation) (Figure 1F). The complexity of iron-deficiency anemia opens myriad possibilities as to how *Ryr2* expression changes, but none is direct, as the gene is not HIF regulated nor does it possess an IRE in either its 5′ or 3′ UTR. Moreover, a transgenic mouse that constitutively overexpresses cardiac HIF1α was associated with a positive inotropic effect (41), which implies that HIF1α signaling per se is unlikely to explain the contractile deficit observed in iron-deficient mice. Western blot analyses demonstrated that RyR2 levels in cultured neonatal myocytes are reduced after at least 16 hours of exposure to 3% hypoxia in a HIF-independent manner but not by the depletion of iron with the chelator DFO (Figure 7C). It is possible that *Ryr2* downregulation relates to increased levels of the repressive mark H3K9me3, which associates with regions of the *Ryr2* gene in hypertrophied hearts (17), although this is not the only histone mark found to associate with this gene (19). This inference would be consistent with the effects of iron and oxygen depletion on KDM4A, the enzyme acting on trimethylated H3K9 (12, 13), shown to be increased in iron-deficient mice (Figure 1I).

The decrease in SERCA activity in iron-deficiency anemia was manifested as a right-shift in its Ca^2+^-activation curve (Figure 5F). It is noteworthy that this shift in activation was not evident from measurements of the time for Fluo3 fluorescence to recover to 50% (Figure 3D). An explanation for this is that a near-parallel shift in an activation curve will not necessarily change the time constant of recovery, if this progresses toward a raised baseline; this conundrum highlights some circumstances that limit the resolving power of nonratiometric dyes. The kinetic remodeling of the Ca^2+^ dependence of SERCA is best described in terms of a 1.6-fold increase in the apparent Ca^2+^ binding constant (K_m_). Raising the K_m_ of SERCA reduces Ca^2+^ sequestration into the SR, thus allowing baseline [Ca^2+^] in the cytoplasm to attain a higher level, as observed in myocytes from iron-deficient mice (Figure 5D). Suppressed SERCA activity is consistent with the faster Ca^2+^ wave velocity (i.e., permitting the cytoplasmic Ca^2+^ signal to diffuse farther) (Figure 6G). In iron deficiency, the concurrent decrease in RyR2 and SERCA activities maintains the pump-leak balance close to control levels, which explains the unaltered diastolic SR Ca^2+^ loading in iron-deficient mice. However, the decrease in RyR2 activity reduces fractional release, which underpins the fall in CaT amplitude. Dual RyR2-SERCA remodeling in iron-deficiency anemia highlights the well-established notion that modulating RyR2 activity alone cannot stably change CaT amplitude (35). This is because an isolated decrease in RyR2 activity would, initially, decrease CaT amplitude but also increase the residual [Ca^2+^] remaining in the SR. As the SR load increases over time, the CaT amplitude will return toward control levels. By also affecting SERCA activity, iron deficiency is able to produce a stable change to CaT amplitude and, hence, contraction. The model proposed herein was verified by mathematical modeling, which demonstrated that the observed changes at transporter level are sufficient to stably change the CaT time course (Figure 6I).

At the molecular level, lower Ca^2+^ affinity relates to the 2-fold decrease in PLN phosphorylation at Thr17, a target of CaMKII (Figure 6, E and F) (28, 29, 31). Acute treatment with ISO, which activates cAMP/PKA signaling and greatly increases overall PLN phosphorylation, collapsed the difference between CaTs measured in iron-deficient and control animals (Figure 3, E and F), implying that a change in baseline PLN phosphorylation underpins SERCA remodeling observed in iron-deficiency anemia. In cultured neonatal myocytes, PLN became dephosphorylated at Thr17 under 3% hypoxia but not in response to treatment with DMOG or DFO under a normoxic environment (Figure 7D), arguing for a metabolic rather than transcriptional mechanism. This change produced a slowing of the recovery phase of the CaT imaged in myocytes cultured for 24 hours in hypoxia (Figure 7, E and G). These findings are consistent with previous reports showing Thr17 dephosphorylation in stop-flow ischemia (30, 31) due to a decrease in CaMKII activity (30), possibly triggered by tissue hypoxia. Since CaMKII is also Ca^2+^ sensitive, it is possible that net Thr17 dephosphorylation is secondary to reduced RyR2 Ca^2+^ fluxes. Under such a mechanism, posttranslational SERCA suppression may be an adaptive change to maintain constancy of the SR Ca^2+^ load.

### Implications for human health.

Iron deficiency is the most common medical condition worldwide, affecting approximately 2 billion people (2). However, in the absence of any underlying heart disease, only very severe anemia is likely to cause high-output HF (42). In keeping with this, the magnitude of the contractile deficit in the present study was moderate, and in particular, the reduction in EF did not fall into the range associated with decompensated HF in more severe experimental models, such as transaortic banding.

The clinical importance of the present findings relates far more to patients with preexisting HF. It is well recognized that iron deficiency and anemia are important comorbidities of HF and that they are associated with poor outcomes (3). On their own, these findings cannot distinguish whether iron deficiency/anemia simply act as markers of more severe disease or play some causal role in worsening the cardiac condition. However, large multi-center trials have shown that treating iron-deficient HF patients with i.v. iron is beneficial (6–8), thus indicating at least some degree of causation between the presence of iron deficiency and HF severity. These trial results, however, provide no insight into the underlying mechanisms by which iron deficiency/anemia can influence cardiac function. Thus, the importance of the present study is that it demonstrates that even moderate levels of iron deficiency/anemia significantly affect Ca^2+^ signaling in a manner that is sufficient to impinge on contractile function. Furthermore, these effects on Ca^2+^ signaling were shown to act through 2 molecules previously implicated in the genesis of HF (15), therefore providing an explanation of why iron deficiency patients are more prone to further contractile deficit. Finally, the cellular changes underpinning the contractile deficit in iron-deficient mice were reversed by 2 doses of FCM, thus offering an explanation for the beneficial effect of i.v. iron supplementation inferred from in human clinical trials.

## Methods

### Iron-deficiency anemia model.

At 3 weeks of age, male WT C57BL/6 mice, bred in-house at the University of Oxford in individually ventilated cages, were weaned on an iron-deficient diet (2–5 ppm iron; Teklad, TK99397; Envigo). Since there are sex-related differences in baseline hematocrit and the cycle-dependent changes in body iron stores in females, male mice were used in this study. Age-matched controls received an iron-adjusted diet (200 ppm iron, TK08713). Mice were given i.v. FCM or saline as sham control. Hemoglobin concentration in tail vein blood, collected using a 27-G needle, was monitored with HemoCue device (Radiometer). Protocols were randomized, but blinding was not implemented because the phenotype was identifiable upon bleeding. The inclusion criterion was completion of the dietary protocol (in this study, no animals were excluded). At the end of the protocol, animals were killed humanely by a Schedule I method or anesthetic overdose. Hearts were either snap-frozen in liquid nitrogen for protein measurements or used for Langendorff perfusion.

### Cine-MRI scanning.

MRI was performed using a 11.7 T (500 MHz) vertical bore system. Anesthesia was induced in 3% isoflurane and maintained in 2% isoflurane. Due to the small size of the rodent heart, high field strengths were applied to improve signal/noise ratios. Electrocardiogram (ECG) electrodes were inserted in the forepaws to provide information for gating, necessary for synchronizing acquisition steps for the relatively high cardiac rate in mice. Images were visualized using a Burker console running Paravision 2.1.1. Cardiac-triggered and respiration-gated cine-FLASH images were acquired, and for each animal, 10–11 contiguous 1-mm thick, true short-axis images were obtained, covering the entire heart. Each in vivo protocol was performed in approximately 45 minutes in the vertical position. EDV and ESV were rendered from Z-stacks and used to calculate stroke volume (EDV – ESV) and EF (SV/EDV).

### Adult ventricular myocyte isolation.

Excised hearts were cannulated at the aorta using a blunted 23-G needle, mounted on a Langendorff apparatus. The heart was first perfused with warm Tyrode perfusion solution containing (in mM): 130 NaCl, 5.6 KCl, 3.5 MgCl_2_, 5 HEPES, 0.4 Na_2_HPO_4_, 10 glucose, and 20 taurine (all from MilliporeSigma). The heart was then digested with Tyrode perfusion solution supplemented with 0.1 mM CaCl_2_ (MilliporeSigma), 1 mM collagenase type II (Worthington) and 0.1 mM protease (MilliporeSigma). Following digestion, cells were dissociated by careful mechanical disruption and filtered through a 500-μm cell strainer. Enzymatic activity was quenched by adding 1% BSA (MilliporeSigma) in Tyrode perfusion buffer. Cells were washed once in 0.5 mM CaCl_2_ (Tyrode) and then once in 1 mM CaCl_2_ (Tyrode) prior to use.

### Neonatal ventricular myocytes.

Primary NRVMs were isolated from 1- to 2-day-old Sprague-Dawley rats (Charles River Laboratories). Pups were culled in accordance with a Schedule 1 method, and their hearts were excised and washed twice in ice-cold 1× ADS buffer containing (in mM): 106 NaCl, 5.3 KCl, 20 HEPES, 5 glucose, 0.4 MgSO_4_, and 0.8 NaH_2_PO_4_. In fresh 1× ADS buffer, the ventricles were finely minced and digested with 0.45 mg/ml collagenase type A (Roche Diagnostics) and 1.25 mg/ml pancreatin (MilliporeSigma) for 5 minutes at 37°C with gentle stirring. The tissue was left to settle, supernatant was discarded, and the tissue was digested for a second time for 8 minutes at 37°C. Enzymatic activity was quenched using newborn calf serum (MilliporeSigma). Cells were pelleted at 1,250 rpm, resuspended in M1 medium: 80% DMEM (MilliporeSigma, D7777) supplemented with 24 mM NaHCO_3_ and 20% M199 (MilliporeSigma, M4530). Complete M1 medium was supplemented with 10% horse serum (MilliporeSigma) and 5% heat-inactivated newborn calf serum (Thermo Fisher Scientific). Cells were preplated on uncoated petri dishes for 2 hours at 37°C. The supernatant was collected and spun at 1,250 rpm for 5 minutes, and the pellet was resuspended in fresh M1 medium. To obtain lysates for Western blotting, cells were plated on fibronectin-coated 6-well plates at approximately 500,000 cells per well in M1 media. For imaging experiments, cells were plated on fibronectin-coated 4-well chambered coverslips (ibiTreat μ-slide; Ibidi, 80426) at approximately 60,000 cells/cm^2^ in M1 media. On day 1 after isolation, cells were serum starved (0%) and maintained in media containing 1:100 ITS (Insulin Transferrin Selenium; Invitrogen). Forty-eight hours (western blotting) or 72 hours (imaging) after isolation, cells were treated for 16 or 24 hours with hypoxia in a chamber at 3% O_2_ or incubated in normoxia in the presence of 1 mM DMOG (MilliporeSigma), 50 μM DFO (MilliporeSigma), or no drug (control).

### Western blotting and immunofluorescence.

Cell lysate was prepared from crushed, frozen heart tissue using RIPA buffer or 8 M urea lysis buffer (for histone blots) supplemented with complete protease inhibitor. Protein lysate (100 μg) was loaded for SDS-PAGE. Membrane was blocked in primary antibody overnight at 4°C and then in HRP-conjugated secondary antibody. For immunofluorescence, isolated cardiomyocytes were plated on to laminin-coated plates and fixed in ice-cold 4% paraformaldehyde. Cells were permeabilized in 0.3% triton-X (MilliporeSigma), blocked first in primary antibody overnight at 4°C and then in secondary antibody and DAPI for nuclear staining. Cells were imaged on a Zeiss LSM 700 confocal microscope at 40× magnification. Densitometry was performed by numerically integrating below the intensity curve within a consistent frame, after offsetting the background. Fast Fourier transform was performed in ImageJ (NIH).

### Transmission electron microscopy (TEM).

Fresh samples were fixed in 2.5% glutaraldehyde (MilliporeSigma) made in 1× PBS, overnight at 4°C. The fixative was removed and replaced with a 0.25% glutaraldehyde solution and kept at 4°C until further processing. Tissues were rinsed 5 times in 1× PBS, 15 minutes for each wash, and were then fixed in 1% osmium tetroxide (OsO_4_; TAAB Laboratories) in 1× PBS at 4°C for 2 hours with gentle rotation. Samples were rinsed 5 times in ddH_2_O, 15 minutes for each wash, and fixed in 0.2% uranyl acetate (Agar Scientific) overnight at 4°C in the dark. Samples were washed once in ddH_2_O for 10 minutes. For dehydration of specimen, samples were washed, for 15 minutes at 4°C, in 30%, 50%, 70%, 80%, 90%, and 95% EtOH. Samples were then incubated 3 times in 100% dry EtOH at 4°C — 30 minutes for each incubation step. For resin infiltration, samples were incubated in 2:1 100% dry EtOH/TAAB low viscosity (TLV) resin (TAAB Laboratories) for 2 hours, 1:1 100% dry EtOH/TAAB TLV resin for 3 hours, and 1:2 100% dry EtOH/TAAB TLV resin for 2 hours with gentle rotation. Samples were then incubated in 100% TAAB TLV resin for 48 hours at room temperature. During this time, the resin was changed every 8 hours. Tissue pieces were transferred to Beem capsules filled with fresh 100% TAAB TLV resin and polymerized overnight at 60°C. Samples were sectioned on the Leica UC7 ultramicrotome. Resin blocks were placed face up using a glass knife in preparation for ultra-thin sectioning. For ultra-thin sectioning, 90-nm thin sections were cut using a diamond knife (Diatome) and transferred to a 200 mesh Cu grid (TAAB Laboratories). Between 4 and 6 sections were cut per sample block. Prior to imaging, grids were poststained with Reynold’s lead citrate for 5 minutes, washed 3 times in ddH_2_O, and air-dried overnight. TEM imaging was performed at 120 kV on the FEI Tecnai 12, and images were acquired using the Gatan OneView CMOS camera with Digital Micrograph 3.0 software.

### Quantitative PCR.

cDNA was reverse transcribed from total RNA using the Expand Reverse Transcriptase kit (Roche Diagnostics) and poly dT primers (Invitrogen), following manufacturer’s instructions. For quantitative PCR (qPCR), 25 μg of cDNA was used as a template, along with either the Taqman Fast Universal or PowerUp SYBR Green master mix (Thermo Fisher Scientific). *Gapdh* (SYBR green assays) or *Actb* (Taqman assays) were used as housekeeper genes. Taqman assays included *Glut1* (Mm00441480), *Glut4* (Mm00436615). The primers used were: *Eno1:* forward 5′-TAGGGTCCGGGCCTCGAT-3′, reverse 5′-TGTCTCGGTTACTAGGCCTGC-3′; *Ryr2:* forward 5′-CGAGGATGAGATCCAGTTCC-3′, reverse 5′-CAAATCCTTCTGCTGCCAAG-3′; *Cacna1c:* forward, 5′-AGCAAGAACCACTGCGGAT-3′, reverse 5′-GAAGAAATGCAGCAACAGCC-3′; *Slc8a1:* forward 5′-TTGAGGACACCTGTGGAGAG-3′, reverse 5′-GGGGCTCTCCAATCTCAAT-3′; and *Atp2a2:* forward 5′-GGGCAAAGTGTATCGACAGG-3′, reverse 5′-TCAGCAGGAACTTTGTCACC-3′.

### Fluorescence imaging of adult myocytes.

Two fluorescent Ca^2+^ dyes were used: Fluo3 (Thermo Fisher Scientific, F1242) for nonratiometric imaging (excitation 488 nm/emission >510 mm) and FuraRed (Termo Fisher Scientific, F3021) for ratiometric imaging, executed in either dual emission (excitation 488 nm/emission 600 ± 10 and 685 ± 25) or dual excitation mode (alternating excitation at 490 nm and 435 nm/emission 645 ± 37.5 nm). To measure cell size, myocytes were loaded with cSNARF1 (Thermo Fisher Scientific, C1270), a bright dye (excitation 555 nm/emission 580–640nm). Photolysis of NP-EGTA (Thermo Fisher Scientific, N6803) was performed using a 40 mW Ar-UV laser (351, 364 nm) . Fluo3 imaging (in line-scan mode) and cSNARF imaging (in xy mode) was performed on a Zeiss LSM 700 system. Dual-emission FuraRed imaging was performed on a Leica SP5 system. Dual-excitation FuraRed imaging was performed with a QImaging camera with a CoolLED pE-4000 LED light source. Cells were loaded with the compounds by AM-loading at room temperature for 5 minutes (Fluo3), 10 minutes (cSNARF1, FuraRed), or 15 minutes (NP-EGTA). To measure electrically evoked CaTs, myocytes were superfused at 37°C with solution containing (in mM): 135 NaCl, 4.5 KCl, 20 HEPES, 1 CaCl_2_, 1 MgCl_2_, and 11 glucose (pH 7.4) at 37°C. Probenecid (1 mM, MilliporeSigma) was included to reduce the degree of dye loss due to extrusion. Caffeine (MilliporeSigma) was included at 10 mM in selected solutions. To elicit Ca^2+^ waves, superfusate Ca^2+^ was raised to 5 mM. To measure β_Ca_, myocytes were superfused in 0Na0Ca containing (in mM): 135 N-methyl-D-glucamine (MilliporeSigma), 1 MgCl_2_, 0.5 EGTA, 4.5 KCl, 11 glucose, and 20 HEPES (pH 7.4) at 37°C.

### Fluorescence imaging of neonatal myocytes.

Ca^2+^ imaging in monolayers was performed using Fluo-3 (excitation 488 nm/emission >510 nm). NRVMs were AM-loaded with Fluo-3 (4 μg/100 μl; reconstituted in 7% Pluronic F-127 [MilliporeSigma]/DMSO) for 10 minutes in normal Tyrode (NT) at room temperature and then superfused in HEPES-buffered Tyrode at 37°C containing (in mM): 135 NaCl, 4.5 KCl, 1 MgCl_2_, 1 CaCl_2_, 20 HEPES, 11 glucose, and 1 probenecid; pH was adjusted to 7.4 at 37°C using 4.0 M NaOH. For cells treated under hypoxic conditions, superfusion was performed in NT bubbled with N_2_.

### Electrophysiology.

Adult myocytes were voltage-clamped in whole-cell mode with a borosilicate glass micropipette (resistance 1–1.5 MΩ) containing an internal solution (in mM): 5 NaCl, 120 CsCl (MilliporeSigma), 1.MgCl_2_, 3 CaCl_2_, 10 TEA-Cl, 5 Mg-ATP (MilliporeSigma), 1 Na_2_-GTP, 5 phosphocreatine, 5 BAPTA (MilliporeSigma), and 10 HEPES (pH 7.2) with CsOH at room temperature. To block K^+^ conductance, the superfusion solution contained (in mM): 135 NaCl, 5.4 CsCl, 1 MgCl_2_, 1.8 CaCl_2_, 5 4-aminopyridine (MilliporeSigma), 10.0 HEPES, and 11 glucose (pH 7.4) with NaOH at room temperature. Measurements were performed at room temperature using an Axopatch200B amplifier.

### Inductively coupled plasma mass spectrometry.

Snap-frozen tissue (5–50 mg) was suspended in 1 ml concentrated nitric acid (MilliporeSigma), digested on a CEM SP-D80 Microwave Digestion System for 10 minutes at 180°C, and diluted to 2% acid in ultra-pure ddH_2_O. Inductively coupled plasma mass spectrometry (ICP-MS) was performed on the Perkin Elmer 6100DRC quadrupole system. For calibrations, 0, 0.5, 1, 10, 20, and 100 ng/g iron was spiked into a selected sample. An external iron standard (ICPMS-68-A solution, High Purity Standards) was measured to validate the calibration. Rhodium (1 ng/g) was also spiked into each sample as an internal standard. For each run, a sample of digested concentrated nitric acid was also submitted. Raw ICP-MS measurements were normalized to wet mass.

### Pentra chemical analysis.

The ABX Pentra 400 was used to measure ferritin, transferrin, and iron in the serum of mice. Concentrations were automatically detected using the reagent cassettes (Horiba) Ferritin 2 CP), Transferrin CP, and Iron CP.

### Antibodies.

Rabbit-Ca_v_1.2 (ACC-003; Alomone Labs), rabbit-RyR2 (MA3-916; Thermo Fisher Scientific), mouse-RyR2 (ab2827; Abcam), mouse-SERCA (NB300-581; Novus Biological), rabbit-ser16-PLN (A010-12; Badrilla), rabbit-thr16-PLN (A010-13; Badrilla), mouse-PLN (A010-14; Badrilla), rabbit-HIF1α (NB100-479; Novus Biological), mouse-HIF2α (clone-190b, MilliporeSigma), rabbit-H3K36me3 (ab9050; Abcam), rabbit-H3K9me3 (ab8898; Abcam), rabbit-H3K4me3 (ab8580; Abcam), rabbit-H3 (ab1791; Abcam), mouse-actin (NB100-74340; Novus Biological), goat anti–rabbit-HRP (sc-2030; Santa Cruz Biotechnology Inc.), goat anti–mouse-HRP (sc-2031; Santa Cruz Biotechnology Inc.), goat anti–rabbit-AlexaFluor 488 (ab15007; Abcam), and goat anti–mouse AlexaFluor 594 (11032; Invitrogen).

### Statistics.

Sample size per experiment was chosen based on the number of animals required to confidently detect statistically significant differences. For all in vivo experiments, sample size was at least 6 animals/group; for ex vivo experiments, sample size was at least 3 animals/group. Protocols were randomized, but blinding was not implemented because the phenotype was readily identifiable upon bleeding. For in vivo studies, the inclusion criterion was completion of the dietary protocol: in this study, no animals were excluded. For Ca^2+^ studies, cells that did not properly respond to electrical pacing or caffeine (i.e., arrhythmic cells) were excluded. No data point or dataset was deemed to be an outlier. The number of biological replicates for each experiment is specified in the figure legends, and exact numbers of animals/cells used per experimental condition can be found in Supplemental Table 1. Two-way ANOVA was used in body mass and hemoglobin comparisons. One-way ANOVA was applied in ICP-MS measurements. Unpaired 2-tailed Student’s *t* test was used for Western blots and qPCR measurements. A nested (hierarchical) 1-way ANOVA was used to analyze other datasets (43). Number of replicates and *P* values are stated in each figure legend. All data are plotted as mean ± SEM. Dot plots are plotted as mean and 5–95 percentile. Significant *P* values are indicated by *P < 0.05, **P < 0.01, ***P < 0.001, ****P < 0.0001. Nested ANOVA *P* values can be found in Supplemental Table 2.

### Study approval.

Animal procedures were performed in compliance with Home Office Guidance on the Operation of the Animals (Scientific Procedures) Act of 1986. All procedures relating to animals were approved by the UK Home Office under the Project License 30/3182 and the local Animal Care and Ethical Review Board (AWERB; Oxford, United Kingdom).

## Author contributions

YJC, SLL, PAR, and PS designed research; YJC, KCP, AL, and AAL performed experiments; YJC, KCP, AL, AAL, and PS analyzed data; and YJC, SLL, PAR, and PS wrote the paper.

## Supplementary Material

Supplemental data

Supplemental Table 1

Supplemental Table 2

Supplemental Table 3

## Version 1 02/19/2019

In-Press Preview

## Version 2 04/04/2019

Electronic publication
