# Supplementary material for: Iron-deficiency anemia reduces cardiac contraction by downregulating RyR2 channels and suppressing SERCA pump activity
Source: JCI Insight. 2019 Apr 4;4(7):e125618. doi: 10.1172/jci.insight.125618 (PMC6483648; doi:10.1172/jci.insight.125618)
Supplement: Supplemental data [file jciinsight-4-125618-s125.pdf]

SUPPLEMENTAL MATERIALS

SUPPLEMENTAL FIGURES

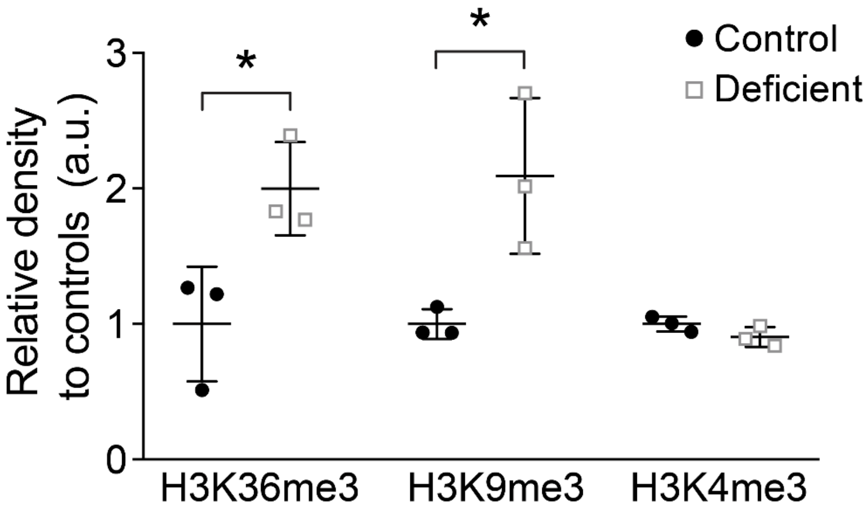

Figure S1. *Quantification of histone methylation.* Densitometric analysis histone 3 trimethylation at lysines 36, 9 and 4 in cardiac lysates from animals on an iron deficient or control diet; n=3 animals/group.

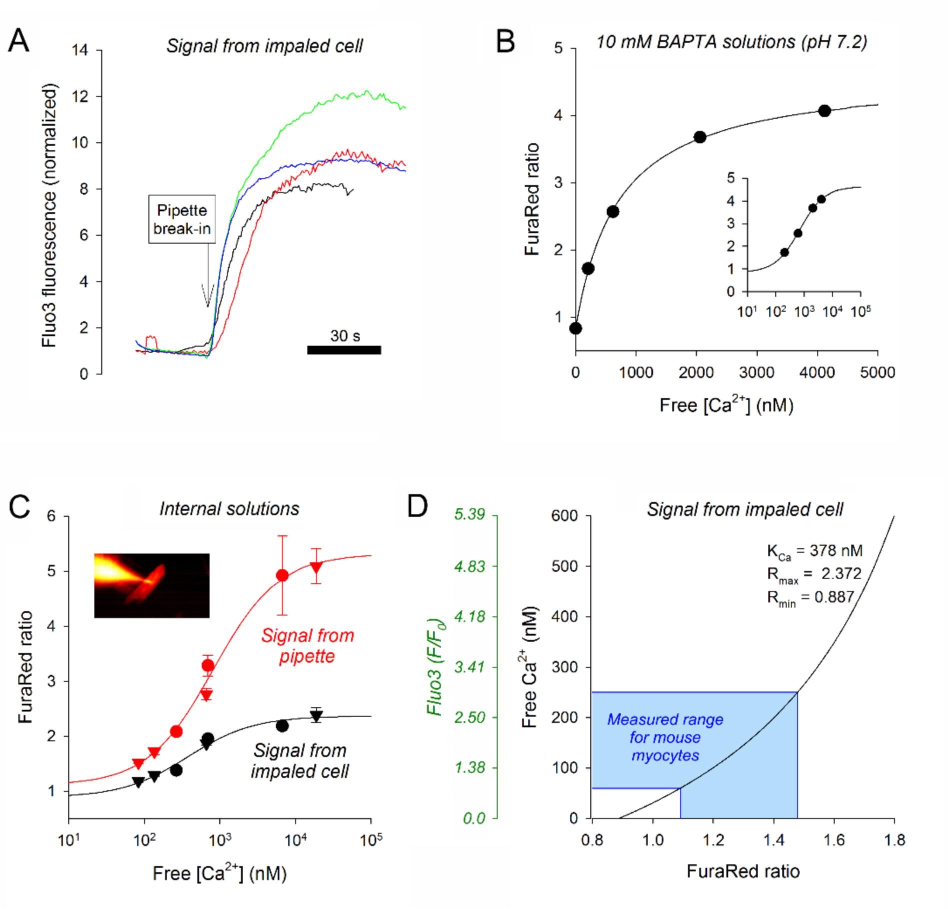

Figure S2. *Calibration of Ca^{2+}-sensitive dyes.* **(A)** Mouse myocyte AM-loaded with Fluo3, and impaled with micropipette containing internal solution with 10 μM Ca^{2+}. Each trace represents a different myocyte. Dialysis of Ca^{2+} into cells produces saturation of Fluo3, thus revealing the dynamic range of the dye. **(B)** FuraRed was calibrated in 10mM BAPTA solutions also containing 10 mM Hepes at pH 7.2, over a range of free [Ca^{2+}] estimated from BAPTA binding kinetics and a Ca^{2+}-binding constant of 160 nM. This standard curve was used to calculate the free [Ca^{2+}] in internal solutions used for filling patch pipettes. **(C)** Internal solutions, containing 50 μM FuraRed (free acid), 140 mM KCl, 10 mM Hepes, 5 mM MgATP, and either 10 mM BAPTA (circles) or EGTA (triangles), plus an amount of $CaCl_2$ added to cover a range of free [Ca^{2+}]. The concentration of [Ca^{2+}] was calculated using the standard curve shown in B. The pipette and impaled cell were imaged on the fluorescence microscopy in FuraRed acquisition mode (dual excitation). The ratio of FuraRed fluorescence in the pipette and in the impaled cell were calculated after the level of fluorescence attained equilibrium i.e. complete dialysis. Mean+/-SEM of 4 cells from 3 animals. Data were best fitted to a Grynkiewicz-type curve. **(D)** The best-fit calibration curve for cytoplasmic free [Ca^{2+}] and its corresponding FuraRed ratio. The region shaded blue shows the physiological range in mouse ventricular myocytes, that includes diastolic and systolic Ca^{2+}. Over this range, FuraRed ratio is near-linearly related to Ca^{2+} and well below the saturating ratio. For comparison, the y-axis is also plotted as the Fluo3 F/F_0 ratio, predicted for a binding constant of 830 nM.

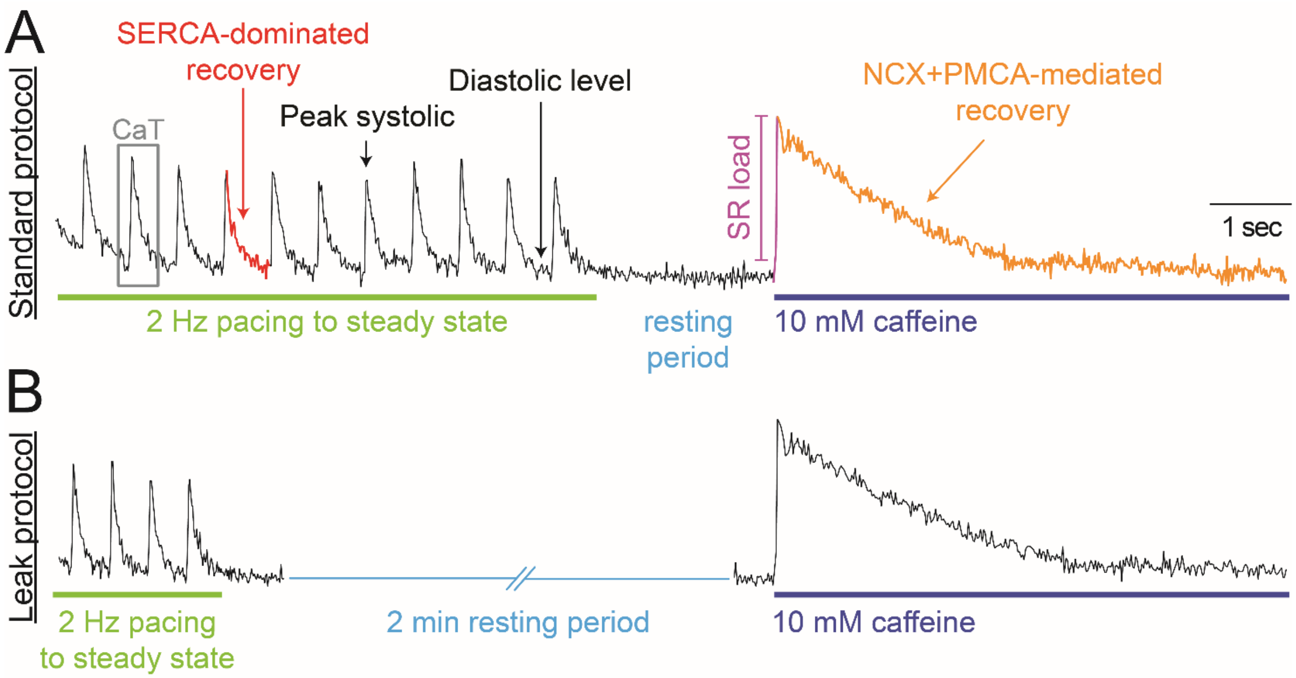

Figure S3. *FuraRed Ca^{2+} imaging protocol.* **(A)** Protocol for interrogating diastolic and systolic [Ca^{2+}], the activity of Ca^{2+} handling proteins, and the SR Ca^{2+} load in FuraRed-loaded myocytes. **(B)** Protocol modified to implement a 2-min resting period before caffeine release for measuring diastolic SR Ca^{2+} leak. The cell was monitored during the rest period for contractile activity (an exclusion criterion).
